# Stroke Mortality and Stroke Hospitalizations: Racial Differences and Similarities in the Geographic Patterns of High Burden Communities Among Older Adults

**DOI:** 10.5888/pcd21.230339

**Published:** 2024-04-18

**Authors:** Kirsten Evans, Michele Casper, Linda Schieb, David DeLara, Adam S. Vaughan

**Affiliations:** 1 Division for Heart Disease and Stroke Prevention, National Center for Chronic Disease Prevention and Health Promotion, Centers for Disease Control and Prevention, Atlanta, Georgia

**Figure:**
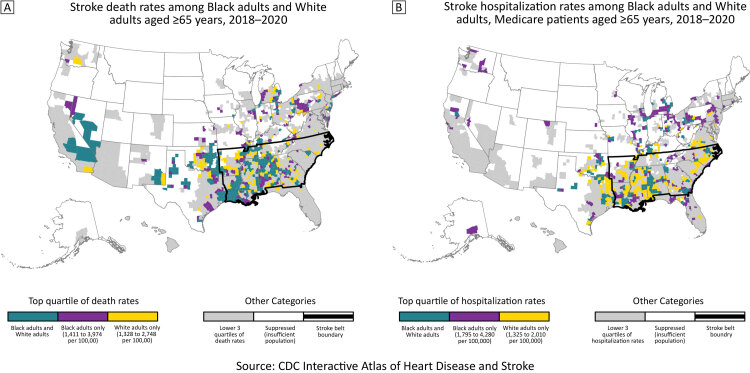
The 2 maps show the top quartiles of county-level race-specific stroke death rates (Map A) and hospitalization rates (Map B) among Black and White adults aged ≥65 years during 2018–2020. Source: The Interactive Atlas of Heart Disease and Stroke (4).

## Background

Geographic patterns of stroke deaths in the US are well documented for Black adults and White adults. Both populations have concentrations of high stroke death rates in the South, commonly known as the Stroke Belt, but geographic patterns of stroke morbidity rates are undocumented (1,2). US stroke incidence data are available only through cohort studies (3). However, county-level stroke hospitalization data can be examined nationally among Medicare beneficiaries, providing a measure of stroke morbidity among older US adults.

Documenting geographic patterns by race for both stroke mortality and hospitalization rates provides a more comprehensive understanding of stroke among Black older adults and White older adults. Additionally, these results provide information for tailoring stroke prevention and treatment programs and policies to communities’ needs. In this study, we compared county-level patterns of stroke death rates (Map A) and stroke hospitalization rates (Map B) for 2018–2020 for Black adults and White adults aged 65 years or older.

## Data and Methods

Three-year average stroke hospitalization and death rates per 100,000 population for 2018–2020 among US non-Hispanic Black adults and non-Hispanic White adults aged 65 years or older were acquired from the Interactive Atlas of Heart Disease and Stroke (hereinafter, Atlas) (4). The Atlas obtains stroke hospitalization data from the Centers for Medicare and Medicaid Services Medicare Provider Analysis and Review file, Part A, and stroke death counts and total population sizes from the National Center for Health Statistics’ National Vital Statistics System. Stroke is defined by *International Classification of Diseases, 10th revision*, codes I60–I69, as the underlying cause of death and principal diagnosis for hospitalizations (5). Rates were age-standardized (using the 2000 US standard population) and spatially smoothed using a local empirical Bayes algorithm (6). Race-specific county rates that did not meet the data suppression criteria for reliability for the Atlas were not included (7).

We calculated quartiles of stroke death rates and hospitalization rates for Black older adults and White older adults separately and created maps comparing geographic patterns of counties in the top quartile for each race. For each county, we also calculated absolute and relative Black–White disparities in stroke death and hospitalization rates. To facilitate comparison between races, we restricted the analysis to counties with reliable stroke death rates (N = 1,679) or hospitalization rates (N = 1,453) for both races (7). Maps showing stroke death and hospitalization rates for all counties are available online (8). We used R version 4.3.1 (R Foundation) (9).

## Highlights

Stroke death rates in the top quartile for both Black adults and White adults aged 65 years or older overlapped considerably: 63% of counties were in the top quartile for both Black older adults and White older adults (teal counties, Map A). Counties in the top quartile of stroke death rates for both populations were concentrated in the western Stroke Belt, Oklahoma, and Texas. Median county-level stroke death rates were 1,214 and 1,155 deaths per 100,000 for older Black and White adults, respectively. The median county-level absolute Black–White disparity in stroke death rates was 61.5 deaths per 100,000 population; the median relative disparity was 1.1.

For stroke hospitalization rates, 44% of counties in the top quartile for Black older adults and White older adults overlapped (teal counties, Map B). In contrast to stroke death rates, counties in the top quartile of stroke hospitalization rates for Black older adults were in the Midwest, Northeast, and South. Counties in the top quartile for White older adults were concentrated in the Stroke Belt — specifically the Mississippi Delta region and into Oklahoma and Texas. The supplemental maps show the full distributions of rates by race (Appendix). Median county-level stroke hospitalization rates were 1,590 and 1,120 hospitalizations per 100,000 for older Black and White adults, respectively. The median county-level absolute Black–White disparity in stroke hospitalization rates was 410 hospitalizations per 100,000, and the median relative disparity was 1.4. 

## Action

Examining geographic patterns of morbidity and mortality rates improves our understanding of the disproportionate burden of stroke across race and geography. Comparing these geographic patterns by race shows notable differences. The historically dominant pattern for the Stroke Belt prevails for stroke mortality rates among Black and White older adults and for stroke hospitalization among White older adults. However, concentration of the highest stroke hospitalization rates in the Midwest and Northeast for Black older adults raises questions about contributors to racial differences in these geographic areas.

Stroke hospitalization rates represent underlying stroke incidence and hospital utilization (10). Understanding factors contributing to concentrations of high stroke hospitalization rates in the Midwest and Northeast for Black older adults requires more closely studying racial patterns in incidence and stroke hospitalization in these communities. Stroke incidence is higher among Black older adults than White older adults, and the magnitude of difference decreases with age (3). Additionally, stroke hospitalization is influenced by stroke literacy and health beliefs of individuals and medical professionals, which affect patients’ trust in the health care system, likelihood to seek care, and likelihood of being admitted to the hospital, many of which are affected by racial discrimination (10,11). Thus, counties with high hospitalization rates and low mortality rates may suggest a health care system with high-quality stroke care. However, low hospitalization rates and high mortality rates suggest that some stroke patients die outside of hospitals. Geographic differences in stroke hospitalizations may also be shaped by state-level policies establishing stroke systems of care and hospital protocols (12,13).

These findings highlight the patterns of counties where stroke burden is greatest for both Black older adults and White older adults. Public health professionals and partners can use these maps to explore and address local conditions driving stroke burden in those communities. Other resources provide valuable information, including the Atlas, materials from the Paul Coverdell National Acute Stroke Program, and the American Heart Association Get With the Guidelines (4,14,15). Meaningfully reducing the burden of stroke in the US may advance by 1) focusing on counties where stroke death rates and hospitalization rates are high for Black older adults and White older adults, and 2) tailoring programs and policies to the needs of those communities.

## Figures and Tables

**Figure:**
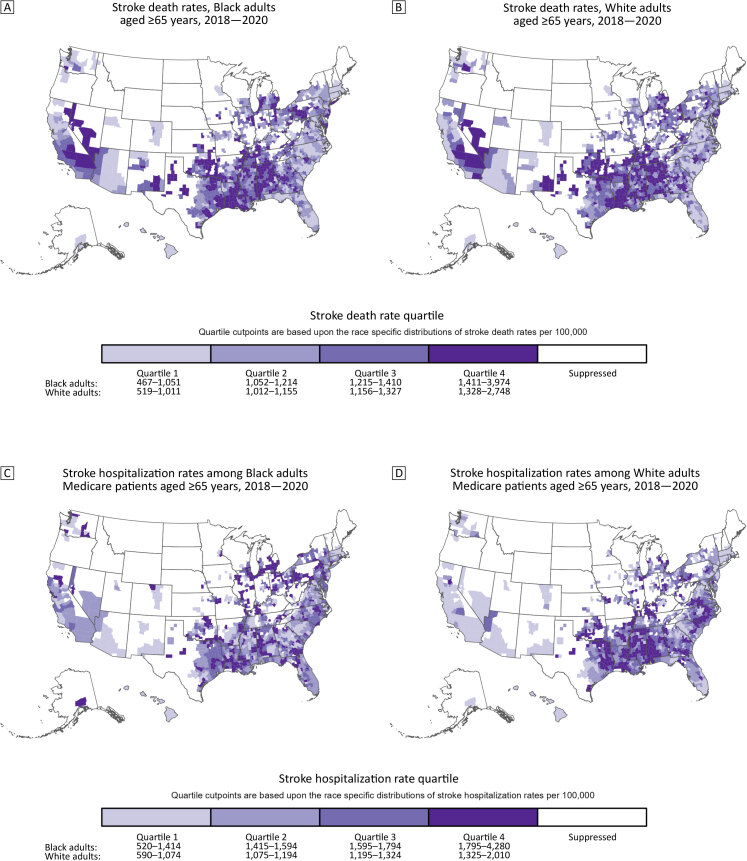
The top maps show county-level stroke death rates for Black adults (Map A) and White adults (Map B) aged ≥65 years, and the bottom maps show county-level stroke hospitalization rates for Black (Map C) and White (Map D) Medicare beneficiaries aged ≥65 years. Quartile cut points are based on the race-specific distributions of stroke death rates or hospitalization rates (respectively) per 100,000 population. Counties had to meet the inclusion criteria to be included on the maps. Source: The Interactive Atlas of Heart Disease and Stroke (4).
